# Catalase in Unexpected Places: Revisiting H_2_O_2_ Detoxification Pathways in Stallion Spermatozoa

**DOI:** 10.3390/antiox14060718

**Published:** 2025-06-12

**Authors:** Ashlee J. Medica, Aleona Swegen, Afshin Seifi-Jamadi, Kaitlin McIntosh, Zamira Gibb

**Affiliations:** Discipline of Biological Sciences, School of Environmental and Life Sciences, College of Engineering Science and Environment, University of Newcastle, Callaghan, NSW 2308, Australia; aleona.swegen@newcastle.edu.au (A.S.); zamira.gibb@newcastle.edu.au (Z.G.)

**Keywords:** antioxidants, hydrogen peroxide, catalase, spermatozoa, horse

## Abstract

Oxidative stress plays a critical role in regulating sperm function, yet species-specific antioxidant mechanisms remain poorly understood. This study compared hydrogen peroxide (H_2_O_2_) tolerance in horse and human sperm and investigated the roles of catalase and glutathione peroxidase (GPx) in horses. A H_2_O_2_ dose–response assay (0–2000 µM) showed that horse sperm were significantly more resistant to oxidative damage, with an IC_50_ for progressive motility over 14-fold higher than that of human sperm (391.6 µM vs. 27.3 µM). Horse sperm also accumulated more intracellular H_2_O_2_ without loss of motility or viability. DNA damage assays (Halo and SCSA) revealed H_2_O_2_-induced fragmentation in human but not horse sperm. Enzyme inhibition experiments in horse sperm using 3-amino-1,2,4-triazole (catalase inhibitor) and (1S,3R)-RSL3 (GPx inhibitor) at 250 µM H_2_O_2_ showed that catalase inhibition severely impaired motility and increased intracellular H_2_O_2_ > 100-fold, while GPx inhibition had a milder effect (~5-fold increase). Immunocytochemistry localized catalase to the sperm head, particularly the post-acrosomal region, challenging the notion that sperm lack peroxisomes. The dependence of horse sperm on oxidative phosphorylation may drive the need for enhanced antioxidant defenses. These findings reveal species-specific oxidative stress adaptations and highlight catalase as a key antioxidant in equine sperm.

## 1. Introduction

Mammalian spermatozoa are prolific generators of reactive oxygen species (ROS), deriving from sources such as sperm mitochondria (due to oxidative phosphorylation; OXPHOS), cytosolic L-amino acid oxidases, and plasma membrane nicotinamide adenine dinucleotide phosphate (NADPH) oxidases. These ROS are natural by-products of cellular metabolism and play crucial roles as signaling molecules at low concentrations, particularly in processes essential for sperm maturation [1,2]. During early-stage capacitation, superoxide (O_2_^•−^) and nitric oxide (NO^•^) activate adenylyl cyclase, leading to the production of cAMP [3,4]. This cAMP activates protein kinase A (PKA), which is critical for supporting the phosphorylation of tyrosine residues on numerous proteins, a process associated with capacitation across various species [1,5,6,7,8].

**Figure 1:**
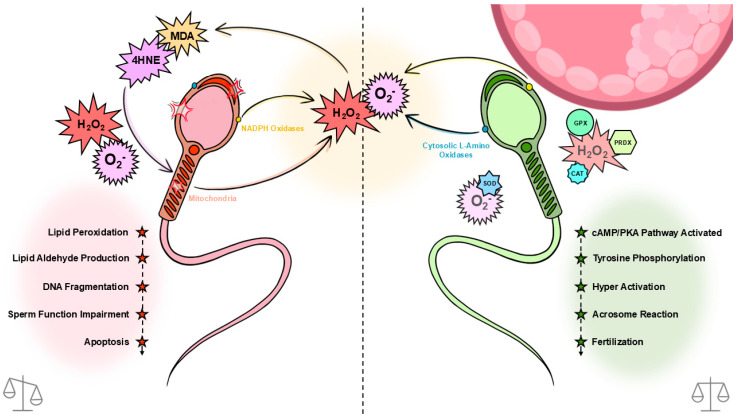
Sperm oxidative stress and protection mechanisms. Primary sources of reactive oxygen species (ROS) in spermatozoa include the mitochondria, cytosolic L-amino acid oxidases, and plasma membrane NADPH oxidases. The ROS generated through these pathways are involved in signalling pathways that regulate sperm function during capacitation, such as tyrosine phosphorylation and the acrosome reaction (right). When the balance between ROS generation and antioxidant defenses—which include glutathione peroxidase (GPx), superoxide dismutase (SOD), catalase (CAT), and peroxiredoxins (PRDXs) is disrupted, oxidative stress ensues (left). This results in lipid peroxidation, the generation of toxic lipid aldehydes such as 4-hydroxynonenal (4HNE) and malondialdehyde (MDA), and, subsequently, oxidative DNA damage. This ultimately leads to DNA fragmentation, impairment of cell functions and apoptosis.

Inversely, at higher concentrations, ROS pose a significant risk to sperm function and fertility. Intracellular antioxidants such as glutathione peroxidase (GPx), superoxide dismutase (SOD), catalase, and peroxiredoxins (PRDXs) assist in protecting the cell against oxidative damage by scavenging ROS. Once these antioxidant defenses are depleted, oxidative stress may lead to lipid peroxidation, ultimately causing DNA damage in sperm cells ([9]; Figure 1). Spermatozoa are particularly vulnerable to oxidative damage due to their membrane’s high proportion of polyunsaturated fatty acids (PUFAs), particularly docosahexaenoic acid [10,11]. These PUFAs provide the membrane fluidity required to engage in membrane fusion events associated with fertilisation, particularly the acrosome reaction and sperm–egg fusion.

Different species exhibit varying sensitivities to reactive oxygen species (ROS), which can be hypothesised as an attribute to their reliance on different methods of ATP production. Species such as horses [12] and boars [13] primarily utilize OXPHOS, a metabolic pathway that generates significant amounts of ROS as metabolic by-products [14]. Consequently, these species typically possess enhanced antioxidant defenses [15]. In contrast, species like bulls and guinea pigs utilize glycolysis and OXPHOS equally [16,17], whereas species such as humans and mice predominantly rely on glycolysis alone [16,18], resulting in lower ROS production. This variation suggests that species relying on OXPHOS, like horses and boars, may require more robust antioxidant defenses to counteract higher ROS levels compared to species that rely more on glycolysis [15].

This delicate balance between ROS-mediated signalling and oxidative damage highlights the importance of understanding ROS dynamics in male reproductive health. Our study aims to compare the sensitivity of spermatozoa from two species to H_2_O_2_, each using different ATP production methods: horse (OXPHOS) and human (glycolysis). Through this comparison, we aim to uncover species-specific antioxidant strategies for mitigating H_2_O_2_ production, as H_2_O_2_ is the most pernicious of the ROS, being membrane permeable. Additionally, we aim to investigate the impact of H_2_O_2_ insult on sperm functionality and explore the relative contributions of catalase and glutathione peroxidase in maintaining ROS homeostasis.

Catalase plays a central role in abolishing H_2_O_2_ in somatic cells, rapidly converting it into water and oxygen, thereby preventing cellular damage [19]. However, its presence and functional relevance in spermatozoa remain controversial. Traditionally, spermatozoa were thought to lack catalase due to the absence of peroxisomes, the primary organelles for catalase localization and activity in most cells [20].

It has been suggested that catalase activity observed in spermatozoa may originate from extracellular sources such as seminal plasma or oviductal fluid. In bulls, for example, catalase was proposed to be acquired from the female reproductive tract, potentially binding to the sperm membrane [21]. More recently, proteomic analyses in several species have identified peroxisomal proteins within spermatozoa [22,23,24,25,26,27,28], renewing interest in the possibility of endogenous catalase expression. Despite this, it remains unclear whether spermatozoa truly express functional catalase or whether the detected proteins reflect remnants of earlier spermatogenic stages or extracellular association. Given catalase’s critical antioxidant role in other cell types, clarifying its presence and function in spermatozoa is essential to understanding how these cells manage oxidative stress, particularly in species like the horse, where reliance on OXPHOS is dominant and ROS production is inherently high.

We hypothesize that horse spermatozoa, due to their reliance on OXPHOS for ATP production, will exhibit greater tolerance to H_2_O_2_ insult compared to glycolytic species like humans, with catalase activity playing a key role in this resistance. While GPx is also essential for H_2_O_2_ detoxification, examining its role alongside catalase will offer deeper insight into the cooperative mechanisms of ROS defence in spermatozoa across species.

## 2. Materials and Methods

### 2.1. Materials

Unless otherwise stated, all reagents used in this study were sourced from Sigma-Aldrich (St. Louis, MO, USA). A modified version of Biggers, Whitten, and Whittingham (BWW) media was employed, comprising 95 mM sodium chloride, 4.7 mM potassium chloride, 1.7 mM calcium chloride dihydrate, 1.2 mM monopotassium phosphate, 1.2 mM magnesium sulfate heptahydrate, 25 mM sodium bicarbonate, 5.6 mM D-glucose, 275 µM sodium pyruvate, 3.7 µL/mL of 60% sodium lactate syrup, 50 U/mL penicillin, 50 µg/mL streptomycin, 20 mM HEPES, and 0.1% (*w*/*v*) polyvinyl alcohol. The final media had an osmolarity of approximately 310 mOsm/kg and a pH of 7.4 [29], and it was used consistently across all experiments unless stated otherwise.

### 2.2. Collection and Preparation of Spermatozoa

#### 2.2.1. Horse

Institutional ethical approval (University of Newcastle Animal Care and Ethics Committee, Approval No. A 2021 139) was secured for the collection of horse spermatozoa from the date 12 June 2024. All experiments were performed using multiple ejaculates from five normozoospermic, reproductively sound Shetland and miniature crossbred pony stallions aged between 5 and 18 years. All stallions were housed on institutionally approved premises and were maintained under consistent husbandry conditions throughout the study. The animals had access to native pasture 24 h per day and were additionally fed a balanced diet once daily. None of the stallions were kept near sites of industrial activity or known environmental toxicant exposure and were considered reproductively normal based on breeding history and semen quality.

Semen collection was performed using a pony-sized Missouri artificial vagina (AV; Minitube, Ballarat, VIC, Australia) fitted with an in-line semen filter. The entire ejaculation was immediately diluted at a 2:1 ratio (extender/semen) using EquiPlus semen extender (Minitube, Ballarat, VIC, Australia). All equipment and extenders were maintained between 37 °C during semen collection and dilution. The extended semen samples were transported to the laboratory in a polystyrene container at room temperature (approximately 20–25 °C).

Upon arrival, a maximum of 6 mL of extended semen was layered over 3 mL of EquiPure (a colloid gradient; Tek-Event Pty Ltd., Rouse Hill, NSW, Australia) and centrifuged at 400× *g* for 20 min. The supernatant was carefully removed, and the sperm pellet was resuspended in 1 mL of BWW, as previously described [30].

#### 2.2.2. Human

Institutional ethical approval (The University of Newcastle Human Research Ethics Committee, approval No. H-2013-0319) was secured for the use of human spermatozoa from the date 4 November 2024. Informed consent was obtained from all subjects involved in the study. Semen samples were obtained from healthy adult male volunteers. All donors had normal semen parameters according to the World Health Organization (WHO) guidelines and reported no history of infertility, chronic illness, or recreational drug use. All donors resided in areas with no known environmental pollution and followed a mixed diet with no specific dietary restrictions or supplementation.

Semen was collected via masturbation after a recommended abstinence period of 2 days. Following liquefaction at 37 °C for 30 min, the semen samples were processed using discontinuous density gradient centrifugation. Motile spermatozoa were isolated by layering the liquefied semen onto a 40/80% percoll gradient and centrifuging at 300× *g* for 20 min. The resulting pellet was washed in BWW, centrifuged again for 10 min at 300× *g*, and resuspended in BWW for use in experiments, as described previously [31].

### 2.3. Sperm Concentration

Sperm concentration was assessed using a NucleoCounter^®^ NC-100™ device (ChemoMetec, Allerod, Denmark). For all experimental procedures, samples were adjusted to a final concentration of approximately 25 × 10^6^ spermatozoa/mL in BWW media.

### 2.4. Sperm Motility

For all species, computer-assisted sperm analysis (CASA; IVOS, Hamilton Thorne, Danvers, MA, USA) was used with the respective program for each species to assess motility parameters, following the settings previously described [30]. A minimum of 200 sperm cells and 5 fields were analysed per sample.

### 2.5. Intracellular Hydrogen Peroxide Concentration (DCF-DA)

Sperm suspensions were pre-loaded with 50 µM (final concentration) 2′,7′-dichlorodihydrofluorescein diacetate (DCF-DA; D399, Thermo Fisher Scientific, Sydney, NSW, Australia) for 30 min at 37 °C. Samples were then centrifuged at 500× *g* for 5 min before removing the supernatant and resuspending in BWW for treatments. After treatments, 400 µL of each sample was transferred to flow cytometry tubes, each containing 5 µL of 1 mg/mL propidium iodide (PI) in Milli-Q water (18.75 µM final staining concentration). Samples were run on BD FACS Canto II with FACSDiva software (version 9.0.1) using a 488 Argon-ion laser, 530/30 band pass (green—for detecting DCF Fluorescence), and 585/42 band pass (red—for detecting PI fluorescence) detection filters. Sperm cells were gated from debris using a forward scatter/side scatter dot plot, and the median DCF signal of the live cells (PI negative) was recorded.

### 2.6. DNA Strand Breaks (Halo Assay)

Following treatment, spermatozoa concentration was adjusted to 10 × 10^6^/mL in BWW, rapidly frozen in liquid nitrogen, and stored at −80 °C until further use. Prior to analysis, the frozen samples were thawed at room temperature and mixed with 1% low-melting point agarose maintained at 37 °C (30 µL of sperm suspension was combined with 70 µL of agarose, yielding a final agarose concentration of 0.7%). A 50 µL aliquot of this mixture was spread evenly onto slides precoated with 0.65% standard agarose. Coverslips were gently applied, and slides were cooled at 5 °C for 5 min. After this, coverslips were removed, and slides were treated with 0.08 N HCl for 7 min at room temperature. Denaturation was then carried out using two solutions: Denaturation Solution 1 (0.4 M Tris, 1% SDS, 50 mM EDTA, 800 mM DTT, pH 7.5) for 10 min, followed by Denaturation Solution 2 (0.4 M Tris, 1% SDS, 2 M NaCl, pH 7.5) for 5 min. Slides were rinsed in 1× TBE buffer for 2 min at room temperature, then dehydrated through a graded ethanol series (70%, 90%, and 100%), each for 2 min, with excess removed between steps. After air-drying, slides were stained with DAPI (1:2000 dilution) for 10 min, washed with PBS, and mounted using 10 µL of Mowiol with coverslip. Fluorescence microscopy was used to assess at least 100 sperm cells per group, categorizing cells based on DNA fragmentation: either with intact DNA (large halos) or fragmented DNA (small or no halos).

### 2.7. Sperm Chromatin Structure Assay (SCSA)

Following treatment, spermatozoa from each treatment were further diluted to 10 × 10^6^/mL using BWW before being snap-frozen in liquid nitrogen and stored at −80 °C until analysis. Following thawing, a 100 µL aliquot of sperm suspension was incubated with 200 µL of acid detergent solution (0.8 N HCl, 0.15 M NaCl, 0.1% Triton X-100; pH 1.2) for 30 s at RT before 600 µL of acridine orange staining solution (0.1 M citric acid, 0.2 M Na_2_PO_4_, 1 mM EDTA, 0.15 M NaCL, 1 M acridine orange) was added. Cells were equilibrated for 2.5 min before immediately being analysed via flow cytometry. Samples were run on BD FACS Canto II with FACSDiva software (version 9.0.1) using a 488 Argon-ion laser and 530/30 band pass (green—for detecting intact, double-stranded DNA) and 670/30 band pass (red—for detecting denatured, single-stranded DNA) filters. Results were reported as the percentage of sperm cells outside of the main population (%DFI), as previously described [32].

### 2.8. Immunocytochemistry

Horse spermatozoa were fixed using 2% paraformaldehyde and incubation at 4 °C for 5 min. Cells were then washed in PBS and stored in 0.1 M glycine/PBS at 4 °C until analysis. At the time of analysis, 50 µL of fixed sperm solution was settled onto poly-L-lysine coated coverslips in a humidified chamber at 4 °C overnight. Coverslip-mounted spermatozoa were washed in PBS for 5 min before cells were permeabilised with 0.2% triton-X in PBS for 15 min at RT. They were then washed in PBS and blocked for 1 h at 37 °C with 3% bovine serum albumin (BSA) and 10% goat serum in PBS. They were then washed with PBS and incubated overnight at 4 °C in a humidified chamber with α-catalase primary antibody (1:100; ab50434; Abcam, Thebarton, SA, Australia) diluted with 1% BSA. They were then washed in PBS and incubated with Alexa Fluor™ 594 donkey anti-goat IgG secondary antibody (1:400; A-11058; Thermo Fisher Scientific, Brisbane, QLD, Australia) for 1 h at 37 °C. Finally, coverslip-mounted spermatozoa were again washed with PBS, and mounted onto microscope slides using Mowiol antifade medium and sealed around the edges with nail varnish. Slides were imaged using fluorescent microscopy at 40× and 100× magnification.

### 2.9. Reverse Transcription PCR

#### 2.9.1. Preparation of RNA from Equine Spermatozoa and Testis Tissue

Following spermatozoa collection and processing as described above, aliquots containing 1 × 10^8^ spermatozoa were washed twice in PBS and pelleted. Testis tissue was donated following routine castrations and stored frozen at −80 °C. TRIzol™ reagent (500 µL; Thermo Fisher, Brisbane, QLD, Australia) was added to spermatozoa and tissue samples and used to homogenise testis tissue. Chloroform (100 µL) was then added to the solution and agitated for 1 min, and the solution was centrifuged at 20,000× *g* for 20 min at 4 °C. The aqueous phase was transferred to a fresh Eppendorf tube, and RNA precipitated by adding an equal volume of isopropanol, along with 5 µL of 2 mg/mL glycogen (Ambion, Austin, TX, USA). The mixture was incubated for 1 h at RT, then centrifuged at 20,000× *g* for 20 min at 4 °C. The RNA pellet was washed with 75% ethanol made up in diethylpyrocarbonate (DEPC) treated water, dissolved in DEPC-treated water, and RNA content was quantified.

#### 2.9.2. Confirmation of Catalase Expression in Equine Spermatozoa and Testis by Reverse Transcription PCR

To confirm the presence of catalase transcripts in spermatozoa and testis tissue, 10 µg of total RNA was reverse transcribed using oligo(dT)_15_ primers (Promega Corporation, Annandale, NSW, Australia) and M-MLV Reverse Transcriptase (Promega). RT-PCR was performed to amplify equine catalase using gene-specific primers: forward 5′-ACTCCCATTGCGGTTCGATT-3′ and reverse 5′-TTGGGTCAAAGGCCAACTGT-3′, designed to yield a 691 bp product. PCR amplification was conducted on an Eppendorf EP Gradient S Thermocycler with the following cycling conditions: initial denaturation at 94 °C for 5 min; 35 cycles of 95 °C for 45 s, 57 °C for 45 s, and 72 °C for 2 min, followed by a final extension at 72 °C for 10 min. PCR products were resolved on 1.5% agarose gels and visualized using a Bio-Rad ChemiDoc™ Imaging System.

### 2.10. Statistics

Data were analysed using GraphPad Prism (version 10.4.2). Normality of distribution was assessed using the Shapiro–Wilk test, with the assumption of normality rejected when *p* ≤ 0.05.

For normally distributed data, repeated measures ANOVA (one- or two-way, depending on the experimental design and whether data were pooled across timepoints) was used to assess differences between treatments. In two-way ANOVAs, if no statistically significant treatment × time interaction was observed, data were pooled across timepoints to isolate the main effect of treatment. When ANOVA indicated a statistically significant treatment effect (*p* ≤ 0.05), Dunnett’s post hoc test was used to identify which treatment groups differed significantly from the control.

For data that were not normally distributed, the non-parametric Friedman test (a repeated-measures alternative to one-way ANOVA) was used to assess differences across treatments. When the Friedman test indicated a statistically significant treatment effect (*p* ≤ 0.05), Dunn’s multiple comparisons test was performed post hoc to identify which treatments differed significantly from the control.

The half-maximal inhibitory concentration (IC_50_) for total and progressive sperm motility was determined using nonlinear regression analysis. Mean values, standard error of the mean (SEM), and sample size (n) were included for each H_2_O_2_ concentration. Data were fitted to a four-parameter logistic dose–response model with a variable slope without log transformation of the concentrations. If motility did not decline to 50% inhibition at the highest tested dose, the IC50 was reported as “>2000 µM H_2_O_2_” to indicate that the inhibitory threshold was not reached within the experimental range. IC50 values were calculated separately for equine and human spermatozoa, and model goodness-of-fit was evaluated by inspecting the 95% confidence intervals and residual plots.

All results are reported as mean ± SEM, and α was set to 0.05.

## 3. Experimental Design

### 3.1. Experiment 1: H_2_O_2_ Dose Response

Spermatozoa from horse (Section 2.2.1) and human (Section 2.2.2) were incubated with increasing concentrations of H_2_O_2_ (0 µM, 250 µM, 500 µM, 1000 µM, and 2000 µM) for 1 h at 37 °C. The H_2_O_2_ concentrations used in this study were chosen based on previous research. Low micromolar to millimolar ranges of H_2_O_2_ have been widely applied to mimic physiological and pathological oxidative stress conditions in vitro [33]. Following incubation, spermatozoa were assessed for intracellular H_2_O_2_ concentration and viability (DCF-DA/PI assay; Section 2.6), and total and progressive motility were assessed using CASA (Section 2.4). For the DCF-DA assay, spermatozoa were preloaded with DCF-DA prior to H_2_O_2_ treatment (Section 2.6). DNA integrity was evaluated using both the sperm chromatin structure assay (SCSA; Section 2.5) and the Halo assay (Section 2.7). All assessments were conducted at 30 min, 1 h, and 2 h post-treatment.

### 3.2. Experiment 2: Antioxidant Inhibition, Detection, and Localisation

Horse spermatozoa were selected for this experiment due to their distinctly higher resistance to oxidative stress observed in Experiment 1, prompting further investigation into their antioxidant defence mechanisms. Horse spermatozoa (Section 2.2.1) were pre-incubated at 37 °C for 30 min with or without the catalase inhibitor 3-amino-1,2,4-triazole (3AT; 25 mM) and the glutathione peroxidase inhibitor (1S,3R)-RSL3 (1 mM; AdooQ Bioscience, Irvine, CA, USA), prior to loading with DCF-DA (Section 2.6). Following pre-incubation, spermatozoa were exposed to either 0 µM (control) or 250 µM H_2_O_2_ for 1 h at 37 °C. Total and progressive motility were analysed using CASA (Section 2.4), while intracellular H_2_O_2_ levels and viability were assessed using DCF-DA and propidium iodide staining, respectively (Section 2.6). Additionally, the presence and localisation of catalase within horse spermatozoa were examined using immunocytochemistry (Section 2.8) and reverse transcription PCR (Section 2.9).

## 4. Results

### 4.1. Experiment 1: H_2_O_2_ Dose Response

All data, except the horse Halo assay (DNA damage) data, were not normally distributed, and so, in these cases, the non-parametric Friedman’s test (for treatment effect) and Dunn’s tests (pairwise comparisons to the control) were utilised instead of the ANOVA and Dunnett’s tests.

Motility: There was no significant interaction between incubation time and H_2_O_2_ dose on either total or progressive motility of spermatozoa from horses or humans, so these data were pooled across time points to show the isolated effect of H_2_O_2_ dose. All pairwise comparisons were made between the control (0 µM H_2_O_2_) and the individual H_2_O_2_ doses.

**Figure 2:**
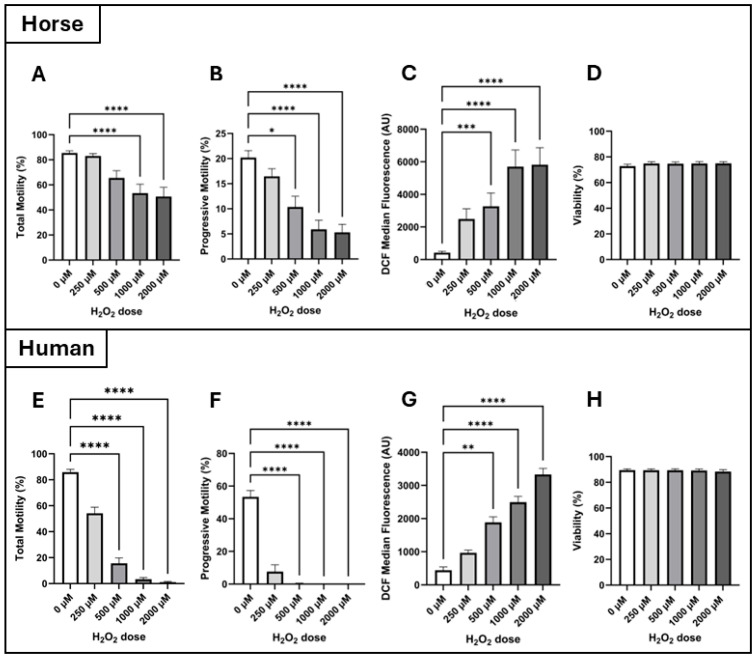
Dose–response effects of hydrogen peroxide (H_2_O_2_) on horse and human spermatozoa. Horse and human spermatozoa were exposed to increasing concentrations of H_2_O_2_ (0, 250, 1000, and 2000 µM), and functional and oxidative stress parameters were assessed at 30, 60, and 120 min post-treatment. (**A**) Total motility, (**B**) progressive motility, (**C**) intracellular H_2_O_2_ levels (assessed via the DCFDA assay and flow cytometry), and (**D**) sperm viability (assessed by propidium iodide staining and flow cytometry) for horse spermatozoa. Equivalent parameters are shown for human spermatozoa. (**E**) Total motility, (**F**) progressive motility, (**G**) intracellular H_2_O_2_ levels (assessed via the DCFDA assay and flow cytometry), and (**H**) sperm viability (assessed by propidium iodide staining and flow cytometry). Data were pooled across time points due to the absence of a time-dependent effect n = 18. Significant difference from the control denoted by * *p* ≤ 0.05, ** *p* ≤ 0.01, *** *p* ≤ 0.001, and **** *p* ≤ 0.0001.

For both horse and human spermatozoa, Friedman’s test revealed an effect of H_2_O_2_ dose (treatment effect) on both total and progressive motilities (*p* ≤ 0.0001). Dunn’s test revealed that in the horse, total motility was lower in spermatozoa incubated with 1000 µM H_2_O_2_ (53.4 ± 7.2%) and 2000 µM H_2_O_2_ (50.7 ± 7.3%) compared to the control (85.5 ± 1.8%; *p* ≤ 0.0001, Figure 2A). In comparison, for human spermatozoa, a significant decrease in total motility was observed at the lower dose of 500 µM compared to the control (15.7 ± 4.2% vs. 85.9 ± 2.2%, respectively, *p* ≤ 0.0001), and this continued to decline at 1000 µM H_2_O_2_ (3.3 ± 1.3%, *p* ≤ 0.0001) and 2000 µM H_2_O_2_ (1.1 ± 0.5%, *p* ≤ 0.0001; Figure 2E). Regarding progressive motility, in the horse, progressive motility began to decline from the 500 µM H_2_O_2_ dose upward compared to the control (control: 20.2 ± 1.4%; 500 µM: 10.4 ± 2.1%, *p* = 0.015; 1000 µM: 5.9 ± 1.8%, *p* ≤ 0.0001; 2000 µM: 5.3 ± 1.6%, *p* ≤ 0.0001, Figure 2B). The same pattern of progressive motility loss was observed in human spermatozoa (control: 53.4 ± 3.9%; 500 µM: 0.4 ± 0.2%, *p* ≤ 0.0001; 1000 µM: 0.1 ± 0.01%, *p* ≤ 0.0001; 2000 µM: 0.02 ± 0.01%, *p* ≤ 0.0001, Figure 2F).

Intracellular H_2_O_2_: Intracellular H_2_O_2_ concentrations were qualitatively assessed using the DCF-DA assay in combination with flow cytometry by measuring the fluorescence intensity of dichlorofluorescein (DCF) in arbitrary units (AUs). There was no significant interaction between incubation time and H_2_O_2_ dose on the intracellular H_2_O_2_ concentration of spermatozoa from horses or humans, so these data were pooled across time points to show the isolated effect of H_2_O_2_ dose. All pairwise comparisons were made between the control (0 µM H_2_O_2_) and the individual H_2_O_2_ doses.

For both horse and human spermatozoa, Friedman’s test revealed an effect of H_2_O_2_ dose (treatment effect) on intracellular H_2_O_2_ concentrations (*p* ≤ 0.0001). Dunn’s test revealed that in both species, intracellular H_2_O_2_ concentration began to increase at the 500 µM dose of H_2_O_2_ compared to the control, and that DCF fluorescence intensity continued to increase with increasing doses of H_2_O_2_ (horse–control: 423.4 ± 84.6 AU; 500 µM: 3267 ± 821 AU, *p* = 0.0006; 1000 µM: 5702 ± 1022 AU, *p* ≤ 0.0001; 2000 µM: 5825 ± 1047 AU, *p* ≤ 0.0001, Figure 2C, and human–control: 441 ± 97 AU; 500 µM: 1885 ± 168 AU, *p* = 0.002; 1000 µM: 2494 ± 174 AU, *p* ≤ 0.0001; 2000 µM: 3333 ± 184 AU, *p* ≤ 0.0001, Figure 2G). At the lowest H_2_O_2_ dose of 250 µM, there was no significant increase in intracellular H_2_O_2_ concentration compared to the control for either species (2488 ± 621 AU and 970 ± 77 AU for horse and human, respectively). Of note, unpaired t-tests revealed that the intracellular H_2_O_2_ concentration of horse spermatozoa was significantly higher than that of human spermatozoa at H_2_O_2_ doses of 250 µM (2488 ± 621 AU vs. 970 ± 77 AU, *p* = 0.021), 1000 µM (3267 ± 821 AU vs. 1885 ± 168 AU, *p* = 0.004), and 2000 µM (5825 ± 1047 AU vs. 3333 ± 184 AU, *p* = 0.025).

Viability: Interestingly, for both species, spermatozoa viability was not affected by H_2_O_2_ treatment across the 2 h incubation time utilised in this study (Figure 2D and Figure 2H for horse and human, respectively).

**Figure 3:**
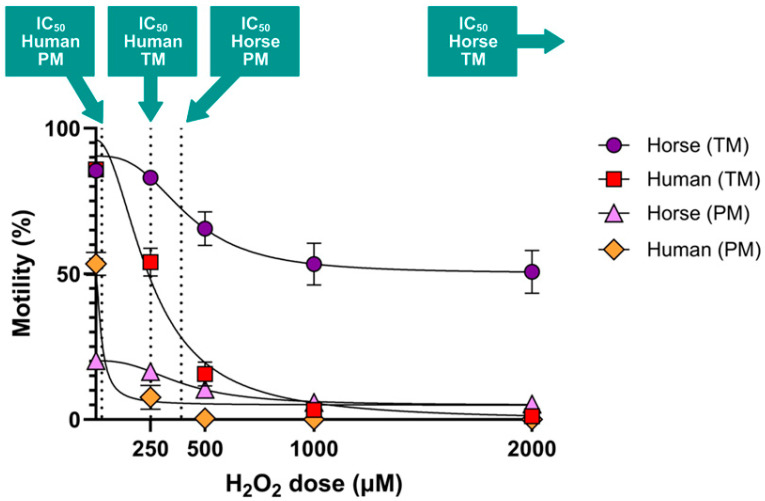
IC_50_ values for hydrogen peroxide-induced reductions in total and progressive motility in horse and human spermatozoa. Spermatozoa were exposed to increasing concentrations of H_2_O_2_ (0, 250, 500, 1000, and 2000 µM), and total and progressive motility were assessed using computer-assisted sperm analysis (CASA). Dose–response curves were generated for each motility parameter, and IC_50_ values were calculated based on nonlinear regression analysis. n = 18.

IC_50_ values: Human spermatozoa are more vulnerable to H_2_O_2_-initiated motility loss compared to horse spermatozoa (Figure 3). Horse spermatozoa total motility did not reach its IC50 at the doses utilised in the present study, so it can only be reported as being > 2000 µM. In contrast, human spermatozoa total motility met its IC50 at 251.9 µM H_2_O_2_. For progressive motility, the IC_50_ of horse spermatozoa was 391.6 µM H_2_O_2_, while the IC50 for human spermatozoa was 27.27 µM H_2_O_2_—more than 14-fold lower than that of the horse.

**Figure 4:**
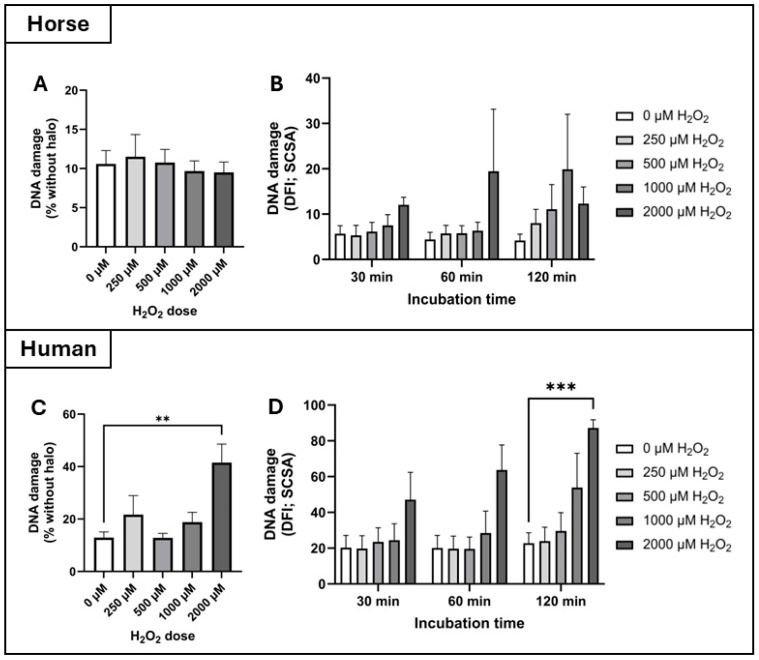
Assessment of hydrogen peroxide-induced DNA damage in horse and human spermatozoa. Spermatozoa were treated with increasing concentrations of H_2_O_2_ (0, 250, 500, 1000, and 2000 µM), and DNA damage was evaluated using two complementary assays. (**A**) Horse sperm DNA fragmentation assessed by the halo assay, expressed as the percentage of spermatozoa without a halo. Data were pooled across time points due to the absence of a time-dependent effect. n = 12; (**B**) horse sperm DNA fragmentation index (DFI) measured using the sperm chromatin structure assay (SCSA) at 30, 60, and 120 min post-treatment. n = 4 per time point; (**C**) human sperm DNA damage assessed by the halo assay. Data were pooled across time points due to the absence of a time-dependent effect. n = 12 (**D**); human sperm DFI measured by SCSA at 30, 60, and 120 min post-treatment. n = 4 per time point. Significant difference from the control denoted by ** *p* ≤ 0.01 and *** *p* ≤ 0.001.

DNA damage: According to the Halo assay, there was no significant interaction between incubation time and H_2_O_2_ dose on DNA damage to spermatozoa from horses or humans, so these data were pooled across time points to show the isolated effect of H_2_O_2_ dose. In the horse, there was no effect of H_2_O_2_ dose on DNA damage as measured by the Halo assay (Figure 4A). In contrast, Friedman’s test revealed an effect of H_2_O_2_ dose (treatment effect) on DNA damage in human spermatozoa (*p* = 0.023) as measured by the Halo assay, but only at the highest H_2_O_2_ dose of 2000 µM (control: 12.9 ± 2.2%; 2000 µM: 41.5 ± 7.1%, *p* = 0.0062, Figure 4C).

According to the sperm chromatin structure assay (SCSA), there was no effect of H_2_O_2_ dose on DNA damage (reported as the DNA fragmentation index; DFI) in horse spermatozoa (Figure 4B). However, there was a significant interaction between incubation time and H_2_O_2_ dose on DFI in human spermatozoa, so the data were analysed at each discrete timepoint (Figure 4D). After 120 min of incubation, Dunn’s test revealed significantly higher levels of DNA damage in human spermatozoa incubated with 2000 µM H_2_O_2_ compared to the control (DFI of 87.2 ± 4.6 vs. 12.3 ± 3.7 respectively, *p* = 0.0005, Figure 4D).

### 4.2. Experiment 2: Antioxidant Inhibition, Detection, and Localisation

The catalase inhibitor 3-Amino-1,2,4-triazole (3-AT) and the glutathione peroxidase inhibitor (1S,3R)-RSL3 (RSL3) were used both alone and with the addition of 250 µM H_2_O_2_ to assess their relative contributions to H_2_O_2_ detoxification in horse spermatozoa. As all resulting data were not normally distributed, they were analysed using Friedman’s tests (treatment effects) and Dunn’s tests to identify whether treatments were significantly different from the control.

**Figure 5:**
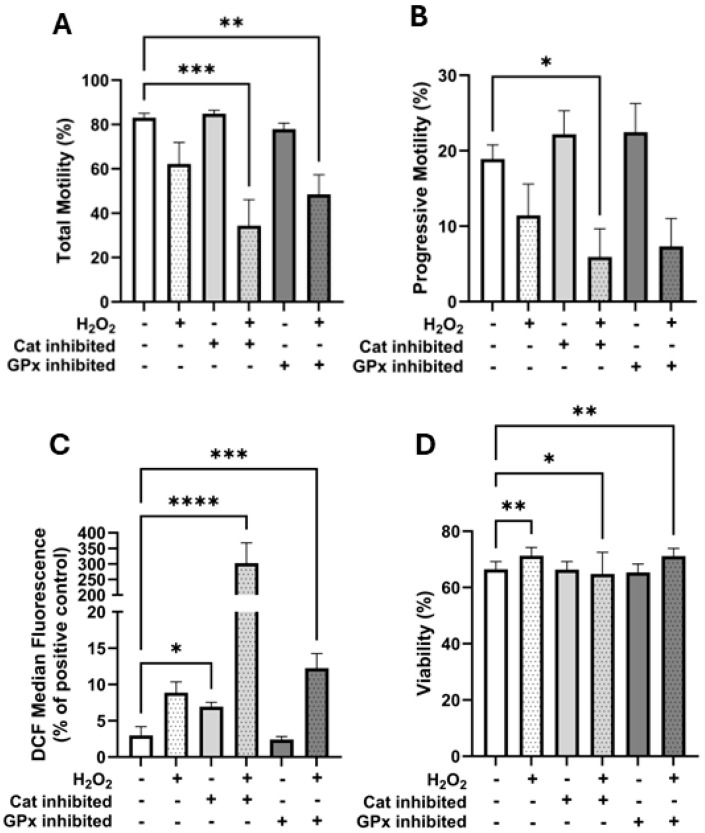
Effects of catalase and glutathione peroxidase inhibition on horse spermatozoa under oxidative stress. Horse spermatozoa were treated with the catalase inhibitor 3-amino-1,2,4-triazole (3AT) or the glutathione peroxidase inhibitor (1S,3R)-RSL3 (RSL3), with (+) or without (−) exposure to 250 µM H_2_O_2_. (**A**) Total motility, (**B**) progressive motility, (**C**) intracellular H_2_O_2_ levels (assessed via the DCFDA assay and flow cytometry), and (**D**) sperm viability (assessed by propidium iodide staining and flow cytometry). n = 10. Significant difference from the control denoted by * *p* ≤ 0.05, ** *p* ≤ 0.01, *** *p* ≤ 0.001, and **** *p* ≤ 0.0001.

Motility: There were significant treatment effects on total motility (*p* ≤ 0.0001), progressive motility (*p* = 0.0015), viability (*p* ≤ 0.0001), and intracellular H_2_O_2_ concentrations (DCF fluorescence; *p* ≤ 0.0001). Total motility was significantly lower than the control only in the treatments which contained both antioxidant inhibitors and H_2_O_2_ (control: 83.1 ± 2.0%; catalase inhibition + H_2_O_2_: 34.4 ± 11.7%, *p* = 0.0004; glutathione peroxidase inhibition + H_2_O_2_: 48.5 ± 8.8%, *p* = 0.0041; Figure 5A). Progressive motility was significantly lower than the control only in the catalase inhibition + H_2_O_2_ treatment (control: 18.9 ± 1.9% vs. catalase inhibition + H_2_O_2_: 5.9 ± 3.7%, *p* = 0.021; Figure 5B).

Intracellular H_2_O_2_: With respect to intracellular H_2_O_2_ concentrations (measured using the DCF-DA assay), the data were normalised and are presented as a percent of the DCF fluorescence of the tert-Butyl hydroperoxide positive staining control for each replicate due to a large degree of variation between replicates (Figure 5C). Compared to the control (3.0 ± 1.2%), intracellular H_2_O_2_ was higher when catalase was inhibited (6.9 ± 0.6%, *p* = 0.043), when catalase was inhibited in the presence of H_2_O_2_ (302.4 ± 65.5%, *p* ≤ 0.0001), and when glutathione peroxidase was inhibited in the presence of H_2_O_2_ (12.2 ± 2.0%, *p* = 0.0004; Figure 5C). H_2_O_2_ treatment alone (8.9 ± 1.5%) and glutathione peroxidase inhibition alone (2.4 ± 0.4%) did not affect intracellular H_2_O_2_ concentrations.

Interestingly, the viability of both the 250 µM H_2_O_2_ treatment (71.3 ± 2.9%, *p* = 0.003) and the glutathione peroxidase inhibition + H_2_O_2_ treatment (71.1 ± 2.8%, *p* = 0.004) was significantly higher than the control (66.5 ± 2.8%), with only the catalase inhibition + H_2_O_2_ treatment having lower viability compared to the control (64.8 ± 7.6%, *p* = 0.025; Figure 5D).

**Figure 6:**
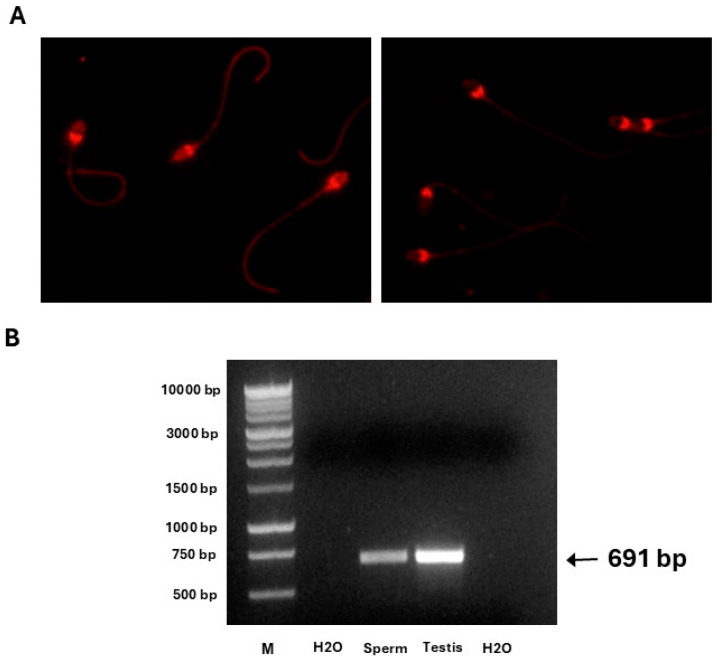
Detection and localisation of catalase in horse spermatozoa and testis. (**A**) Immunocytochemistry (ICC) showing the localisation of catalase in horse spermatozoa, with fluorescence observed predominantly in the post-acrosomal region using a 40× objective. (**B**) Reverse transcription PCR analysis confirming the presence of catalase in both ejaculated horse spermatozoa and testis tissue with a band present at 691 bp for both.

Catalase localisation: To further investigate the source of catalase activity in horse spermatozoa, reverse transcription PCR was performed using RNA extracted from both ejaculated horse spermatozoa and testis tissue (Figure 6). A distinct band corresponding to the expected product size (691 bp) for catalase was observed in both tissue types, confirming the presence of catalase transcripts. Complementing this, immunocytochemistry revealed specific localisation of catalase protein within the sperm head, particularly in the post-acrosomal region (Figure 6).

## 5. Discussion

This is the first study to directly compare the impacts of exogenous hydrogen peroxide (H_2_O_2_) on horse and human spermatozoa, two species that rely on distinct ATP production strategies: OXPHOS and glycolysis, respectively. We hypothesised that horse spermatozoa would demonstrate greater tolerance to H_2_O_2_-induced oxidative stress due to their high mitochondrial activity and reliance on OXPHOS, which likely necessitates more robust antioxidant defenses. Our findings support this hypothesis, demonstrating that human spermatozoa are significantly more susceptible to oxidative insult, as shown by hydrogen peroxide dose–response experiments (Figure 2). The calculated IC_50_ values for progressive motility highlighted this disparity, 27.27 µM for human spermatozoa compared to 391.6 µM for horse spermatozoa, underscoring the greater oxidative resilience of horse spermatozoa (Figure 3). This trend was further supported by DNA damage assays: exposure to 2000 µM H_2_O_2_ significantly increased DNA fragmentation in human spermatozoa (as measured by both the Halo and SCSA assays), whereas horse spermatozoa exhibited no significant increase in DNA damage at any dose of H_2_O_2_ (Figure 4).

At the molecular level, H_2_O_2_ can give rise to highly reactive hydroxyl radicals via Fenton chemistry involving transition metals such as Fe^2+^. These radicals can attack guanine residues and the sugar-phosphate backbone, leading to the formation of lesions such as 8-hydroxy-2′-deoxyguanosine (8-OHdG), single- and double-strand breaks, and chromatin cross-linking [34]. As mature spermatozoa lack the machinery for DNA repair, such oxidative lesions accumulate and may compromise fertilisation, embryo development, or offspring health. Therefore, elevated DNA damage in human sperm under oxidative stress may reflect inadequate antioxidant neutralization. These species-specific differences in DNA damage may also relate to variations in sperm nuclear basic proteins (SNBPs), which help compact and protect sperm DNA. These differences could influence the accessibility of ROS to sperm DNA and subsequent damage. Thus, variation in SNBP content and structure between humans and horses may represent an additional layer of protection in the latter, warranting further comparative investigation.

Interestingly, intracellular H_2_O_2_ measurements revealed a seemingly paradoxical pattern: although both species showed increasing DCF fluorescence with rising H_2_O_2_ concentrations, horse spermatozoa consistently exhibited higher intracellular H_2_O_2_ levels at equivalent doses (Figure 2C,G). This suggests that while horse spermatozoa internalise more H_2_O_2_, they are better equipped to tolerate or neutralise the resulting oxidative stress. Supporting this, inhibition of catalase significantly impaired horse spermatozoa’s ability to manage intracellular ROS and maintain motility (Figure 5). One possible explanation is that horse spermatozoa may have a greater capacity for H_2_O_2_ uptake or accumulation, balanced by more robust antioxidant defenses, particularly catalase activity. Thus, higher intracellular H_2_O_2_ does not necessarily indicate impaired degradation but reflects a species-specific equilibrium between H_2_O_2_ influx and detoxification. Nonetheless, a direct assessment of H_2_O_2_ transport mechanisms and enzymatic degradation kinetics is warranted to fully elucidate these species differences.

The presence of catalase in spermatozoa remains controversial due to the traditional view that spermatozoa lack peroxisomes, the typical site of catalase expression [34]. Nonetheless, multiple proteomic studies across several species have identified peroxisomal proteins in spermatozoa [22,23,24,25,26,27,28]. In human spermatozoa, 1000 proteins have been identified in the sperm tail proteome [23], with a notable proportion related to metabolic functions, including those involved in mitochondrial β-oxidation and peroxisomal activity. Unexpectedly, various peroxisomal proteins were detected, some involved in the oxidation of very long-chain fatty acids, challenging the traditional view of peroxisomal inactivity in spermatozoa. Functional analysis suggested that both mitochondrial and peroxisomal pathways may be active in human spermatozoa and that fatty acid metabolism may contribute more substantially to energy production than previously thought. Nonetheless, although peroxisomal enzymes have been identified, the functional presence of catalase in ejaculated human spermatozoa remains unconfirmed. Catalase was not detected in the sperm tail proteome of Amaral et al.’s study, possibly due to its true absence or its presence at levels below the detection threshold. Additionally, human spermatozoa are still widely considered to rely primarily on glycolysis for ATP generation [18], and intracellular antioxidant defence is thought to depend more heavily on the glutathione peroxidase (GPx) family [35,36]. While catalase activity is known to exist in seminal plasma [37], further work is needed to clarify whether functional catalase is present within human sperm cells themselves.

In contrast, multiple peroxisomal proteins have been identified in horse spermatozoa [28], including enzymes specifically involved in the β-oxidation of very long-chain fatty acids. This provides stronger support for functionally active peroxisomes in horse spermatozoa and suggests that fatty acid metabolism plays a significant role in energy production in this species. These findings are consistent with the known reliance of horse spermatozoa on OXPHOS for ATP production [12] and align with our data showing superior oxidative stress resistance in horse compared to human spermatozoa.

A prior study in bull epididymal spermatozoa reported catalase activity originating from oviductal fluid [21]. However, our study used ejaculated horse spermatozoa, which had been exposed to seminal plasma rather than oviductal secretions. While it is possible that the catalase detected was from seminal plasma [38], our immunocytochemistry results showed clear localisation of catalase within the sperm head (Figure 6A), suggesting an intracellular source. Furthermore, PCR analysis confirmed the presence of catalase in both horse spermatozoa and testis tissue, supporting endogenous expression (Figure 6B). Although we cannot definitively confirm active enzymatic function without specific activity assays, these findings strongly imply that catalase is functionally present in horse spermatozoa and may be linked to broader oxidative metabolic pathways.

To further investigate the role of antioxidant enzymes, we used specific inhibitors targeting catalase and GPx. Catalase inhibition in the presence of H_2_O_2_ significantly reduced total and progressive motility (Figure 5A,B), increased intracellular H_2_O_2_ (Figure 5C), and decreased viability (Figure 5D). In contrast, GPx inhibition led to increased intracellular ROS (Figure 5C) and reduced total motility (Figure 5A) but had a less significant effect on progressive motility and viability (Figure 5B,D). These data suggest that while both enzymes contribute to antioxidative defence, catalase may play a more pivotal role in preserving sperm function under oxidative stress in horse spermatozoa.

It is worth noting that our study did not assess enzyme-specific activity for catalase or GPx due to limitations in current assays. These enzyme activity assays rely on the measurement of the degradation of H_2_O_2_, making it impossible to distinguish the specific individual enzyme contributions. Moreover, we cannot rule out the possibility of incomplete enzyme inhibition under our experimental conditions. The modest effects observed with GPx inhibition may reflect partial inhibition or lower baseline activity.

Finally, the elevated basal antioxidant activity observed in horse spermatozoa may protect against oxidative insult but could also hinder capacitation, a process that relies on transient ROS signaling to activate pathways such as the MAPK cascade, which regulates protein phosphorylation and the acrosome reaction. Excessive antioxidant activity may dysregulate these redox-sensitive pathways, impairing the signaling required for capacitation. This trade-off may help explain the well-documented difficulties in achieving capacitation in equine spermatozoa [39] and the extensive incubation time required to capacitate horse spermatozoa for use in IVF [40]. Interestingly, we also found that low concentrations of H_2_O_2_ (250 µM) were associated with increased sperm viability, both in the standalone H_2_O_2_ group and the GPx inhibition plus H_2_O_2_ group (Figure 5D). This seemingly paradoxical effect may reflect the role of H_2_O_2_ as a modulator of cell signalling, capable of activating kinases and inhibiting proteases to enhance cellular homeostasis in the short term [41,42]. Such biphasic responses are consistent with the concept of oxidative eustress, where ROS serve not only as damaging agents but also as essential signalling molecules at sublethal levels [43].

In addition, exposure to pollutants such as heavy metals, pesticides, and endocrine-disrupting chemicals has been linked to increased oxidative stress in spermatozoa, leading to impaired motility, DNA damage, and altered seminal plasma composition [33,44]. A recent study demonstrates that exposure to environmental toxicants can induce transcriptional responses and alter the structure of SNBP, such as protamines and protamine-like proteins, thereby potentially impairing fertilisation and embryo viability [45]. While our work focuses on species-specific antioxidant defenses, future studies should consider environmental exposures as critical modifiers of sperm oxidative homeostasis and fertility potential.

Further research should also explore proteomic and post-translational modifications in sperm exposed to oxidative stress. Key proteins involved in motility, capacitation, and fertilisation are highly sensitive to oxidative post-translational modifications such as carbonylation and phosphorylation, which can impair their function. Comparative proteomic studies may reveal species-specific adaptations in antioxidant and mitochondrial proteins that contribute to oxidative resilience, offering new targets to improve sperm function. While this study provides novel insights into species-specific oxidative stress responses in spermatozoa, several limitations should be acknowledged. First, we did not directly measure the enzymatic activity of catalase or GPx, limiting our ability to quantify their functional contributions. Although immunocytochemistry and PCR suggest catalase presence in horse sperm, its precise intracellular localization and active enzymatic role within the sperm cytoplasm remain unconfirmed. Second, while we used specific inhibitors, off-target effects or incomplete inhibition cannot be ruled out. Third, we did not assess downstream oxidative stress signaling pathways or post-translational modifications, which could further clarify the molecular impact of ROS. Finally, while our sample sizes were sufficient to detect key differences, the inherent variability in donor background (e.g., genetic and environmental factors) may influence results and should be addressed in future studies with larger, more diverse cohorts.

In conclusion, horse spermatozoa demonstrated significantly greater resilience to oxidative insult, likely reflecting their reliance on OXPHOS and the corresponding need for robust antioxidant defenses. The identification of intracellular catalase, supported by immunocytochemistry and PCR, alongside functional data from enzyme inhibition experiments, suggests that catalase plays a critical role in mediating this protection in horse spermatozoa. In contrast, human spermatozoa, which primarily utilise glycolysis, were more vulnerable to oxidative damage and may rely more heavily on the glutathione peroxidase system for ROS homeostasis. These findings underscore fundamental differences in oxidative metabolism and antioxidant strategies between species and may help explain the unique challenges associated with horse sperm capacitation.

## Data Availability

The raw data supporting the conclusions of this article will be made available by the authors on request.
